# Structure, optical and magnetic properties of new Bi_0.5_Na_0.5_TiO_3_- SrMnO_3−δ_ solid solution materials

**DOI:** 10.1038/s41598-019-54172-4

**Published:** 2019-12-03

**Authors:** Dang Duc Dung, Nguyen The Hung, Dorj Odkhuu

**Affiliations:** 1 Department of General Physics, School of Engineering Physics, Ha Noi University of Science and Technology, 1 Dai Co Viet road, Ha Noi, Viet Nam; 2 Department of Physics, Faculty of Basic and Fundamental Sciences, Viet Nam Maritime University, 484 Lach Tray street, Hai Phong city, Viet Nam; 3 Department of Physics, Incheon National University, Incheon, 22012 Republic of Korea

**Keywords:** Ferromagnetism, Magnetic properties and materials, Spintronics

## Abstract

The new Bi_0.5_Na_0.5_TiO_3_-SrMnO_3−δ_ solid solution materials were fabricated via sol–gel method. The random incorporation of Sr and Mn cations into host lattice of Bi_0.5_Na_0.5_TiO_3_ resulted in structural distortion and influenced on the reduction of the optical band gap from 3.07 eV to 1.81 eV for pure Bi_0.5_Na_0.5_TiO_3_ and 9 mol% SrMnO_3−δ_ solid solution into Bi_0.5_Na_0.5_TiO_3_. The magnetic properties of Bi_0.5_Na_0.5_TiO_3_ materials at room temperature were tuned via compensation of diamagnetic material with weak-ferromagnetism to ferromagnetism with low SrMnO_3−δ_ content and combination of paramagnetism/antiferromagnetism-like and ferromagnetism with higher SrMnO_3−δ_ content solid solution in Bi_0.5_Na_0.5_TiO_3_. The tunable magnetic and optical properties of lead-free ferroelectric materials was promising for their application to green electronic devices.

## Introduction

Bi_0.5_Na_0.5_TiO_3_ materials and their solid solution showed rapid development, especially in the search of high-performance lead-free piezoelectric materials to address human health and environmental protection concerns^1,2^. Bi_0.5_Na_0.5_TiO_3_ material which was firstly fabricated by Smolenskii *et al*. in 1961, is an *A*-site complex perovskite-structured material with random distribution of Bi and Na cation at *A*-site^3^. At room temperature, Bi_0.5_Na_0.5_TiO_3_ materials has a rhombohedral symmetry with Curie temperature (*T*_C_) ~320 °C, remanence polarization (*P*_r_) ~38 μC/cm^2^, high dielectric constant (*ε*_r_ ~694 at 1 kHz) and low dielectric loss (tan*δ* ~0.103) while high coercive field ~7.3 kV/mm, thereby resulting in weak piezoelectric coefficient (*d*_33_) ~73–80 pC/N due to hard to polling treatment^4,5^. The Bi_0.5_Na_0.5_TiO_3_ materials displayed a direct transition optical band gap (*E*_g_~3.01–3.18 eV), depending on the fabrication method^6^. However, the performance properties of lead-free ferroelectric Bi_0.5_Na_0.5_TiO_3_ materials are still not comparable with those of Pb(Ti,Zr)O_3_-based materials in terms of application requirements in electronic devices^7,8^. The high-performance properties of lead-free ferroelectric Bi_0.5_Na_0.5_TiO_3_ materials were recently greatly enhanced by using a solid solution with various compounds containing transition metal such as BiCoO_3_, Bi(Zn_0.5_Hf_0.5_)O_3_, Bi(Mn_0.5_Ti_0.5_)O_3,_ Bi(Co_0.5_Ti_0.5_)O_3_ etc.^4,9–12^. Guo *et al*. reported that BiCoO_3_-modified Bi_0.5_Na_0.5_TiO_3_ materials were exhibited the increasing *d*_33_ values up to 107 pC/N, whereas and the coercive field were reduced to 5.25 kV/mm^4^. The Bi(Zn_0.5_Hf_0.5_)O_3_-modified Bi_0.5_Na_0.5_TiO_3_ materials increased the *P*_r_ and *T*_C_ values to 43.5 μC/cm^2^ and 340 °C, respectively^9^. Bi(Mn_0.5_Ti_0.5_)O_3_ and Bi(Co_0.5_Ti_0.5_)O_3_ solid solution into Bi_0.5_Na_0.5_TiO_3_-based materials resulted in a display a giant electrical field-induced strain coefficient values (d^*^_33_) to 818 pm/V and 600 pm/V, respectively^10–12^. In addition, the *A*-site was modified Bi_0.5_Na_0.5_TiO_3_ materials via Sr as solid solution of SrTiO_3_ in Bi_0.5_Na_0.5_TiO_3_ materials, a large electrical field-induced strain of over 1000 pm/V for low-driving fields (less than 2 kV/mm) was achieved^13^. The solid solution with perovskite-type material containing the transition metal and alkaline cations possibly enhanced the performance of electrical properties of lead-free ferroelectric Bi_0.5_Na_0.5_TiO_3_-based materials.

The observation of room temperature ferromagnetism in pure Bi_0.5_Na_0.5_TiO_3_ materials was promising for the transfer of lead-free ferroelectric material to multiferroic applications in electronic devices^14–17^. Ju *et al*. obtained the room temperature ferromagnetism in nanocrystalline Bi_0.5_Na_0.5_TiO_3_ and its possible origin from Na vacancies located at/near surface of nanograins^14^. Thanh *et al*. also achieved the room temperature ferromagnetism versus diamagnetism in pure Bi_0.5_Na_0.5_TiO_3_ materials^15,16^. Zhang *et al*. predicted the ideal Bi_0.5_Na_0.5_TiO_3_ non-magnetic material; whereas Na or Ti vacancies can induce the magnetism rather than Bi or O vacancies by using ab initio calculations^17^. However, the main problem of magnetism in pure Bi_0.5_Na_0.5_TiO_3_ compounds is low magnetisation (less than 1 memu/g) and strong influence of diamagnetic components, which were raised from empty orbital of 3*d*°-Ti^14–16^. Therefore, for a new Bi_0.5_Na_0.5_TiO_3_-based compound is important for the transfer of the materials to industrial application in smart electronic devices. Simple ideas were used for investigating high ferromagnetism at room temperature; transition metal was used as impurities for substitution at Ti-site in perovskite structure. Room temperature ferromagnetism in Bi_0.5_Na_0.5_TiO_3_ was reported for Co-, Mn-, Ni-, Fe- and Cr-dopants^15,16,18–20^. However, the physical property of room temperature ferromagnetism ordering phenomenal was raised from various parameters, was not well understood. The room temperature ferromagnetism in Mn-, Ni- and Fe-doped Bi_0.5_Na_0.5_TiO_3_ compounds are intrinsic phenomena that resulting from the interaction between magnetic ions through the oxygen vacancies^16,18,20^. It is unlikely that the room temperature ferromagnetism in Cr-doped Bi_0.5_Na_0.5_TiO_3_ compound originated more from oxygen vacancies than the interaction of Cr ions, whereas ferromagnetism in Co-doped Bi_0.5_Na_0.5_TiO_3_ compound was exhibited from magnetic Co clusters^15,19^. The other method was used in tailoring ferromagnetic properties in lead-free ferroelectric Bi_0.5_Na_0.5_TiO_3_ materials that sintered the ferroelectric-ferromagnetic materials as composites, such as CoFe_2_O_4_/Bi_0.5_Na_0.5_TiO_3_ and MgFe_2_O_4_/Bi_0.5_Na_0.5_TiO_3_^21,22^. However, the main problem of the other method is the pole under low-electrical field during high conductivity of spinel and/or interface effect, which results in large leakage current^23^. Recently, we proposed the new method for arching the room temperature of solid solution with alkaline-transition compounds such as MgFeO_3−δ_ or SrFeO_3−δ_^24,25^. In the solid solution, both *A*- and *B*-sites of Bi_0.5_Na_0.5_TiO_3_ compounds were modified by alkaline cation and transition metal ions, respectively, thereby resulting display ferromagnetism with large magnetisation at room temperature and overcoming the single transition metal dopants.

To date, no reports on the use of Mn-based alkaline material as solid solution in Bi_0.5_Na_0.5_TiO_3_ materials are available. Alkaline-earth metal manganese double oxides are interesting materials because oxygen deficiency can be modulated according to their structural, electrical, and magnetic properties^26–30^. Kobayashi *et al*. reported that SrMnO_3_ and SrMnO_2.5_ compound exhibited the cubic and orthorhombic structure^27^. Hexagonal SrMnO_3_ was possibly transformed into the pseudocubic SrMnO_3–δ_ by introducing oxygen vacancies and final reducing state to orthorhombic SrMnO_2.5_ structural^27^. The valence state of Mn near the interface gradually varied from Mn^3+^ to Mn^4+^ over an area of a few atomic layers^27^. Belik *et al*. reported that the polymorphous crystal structures of hexagonal 6H-SrMnO_3_, hexagonal 4H-SrMnO_3_, and cubic SrMnO_3_ have the same chemical composition as SrMnO_3_^28^. Suescun *et al*. reported that oxygen vacancies ordering in oxygen-deficient perovskites SrMnO_3–δ_ compounds were possibly reduced to SrMnO_2.6_ and SrMnO_2.74_ with tetragonal and monoclinic properties, respectively^28^. The 6H-SrMnO_3_, 4H-SrMnO_3_, and cubic-SrMnO_3_ exhibited the antiferromagnetic property with Neel temperatures (*T*_N_) of 235 K, 280 K, and 240 K, respectively^27^. The cubic-SrMnO_3_ exhibited a *G*-type antiferromagnetic structure with *T*_N_ in the range 230–260 K probably because of the small variations in oxygen stoichiometry^28,30^. Rahman *et al*. predicted that SrMnO_2_ is a tetragonal lattice structure with an *A*-type antiferromagnetic conductor^31^. Given the well solid solution of SrMnO_3–δ_ into host Bi_0.5_Na_0.5_TiO_3_ crystal, we expected that the Sr and Mn cations were diffused to randomly incorporate with the host lattice of Bi_0.5_Na_0.5_TiO_3_ crystal to form a solid solution. Thus, the interaction between random magnetic Mn cations at the *B*-site and co-modified by Sr cations at the *A*-site in host Bi_0.5_Na_0.5_TiO_3_ materials was expected to be of phenomenal interest. In this work, the solid solution of (1−*x*)Bi_0.5_Na_0.5_TiO_3_ + *x*SrMnO_3−δ_ compounds was fabricated using sol–gel method. The solid solution SrMnO_3−δ_ in Bi_0.5_Na_0.5_TiO_3_ compound reduced the optical band gap and tunable magnetic properties of host materials.

## Results

### Chemical compositions

**Figure 1:**
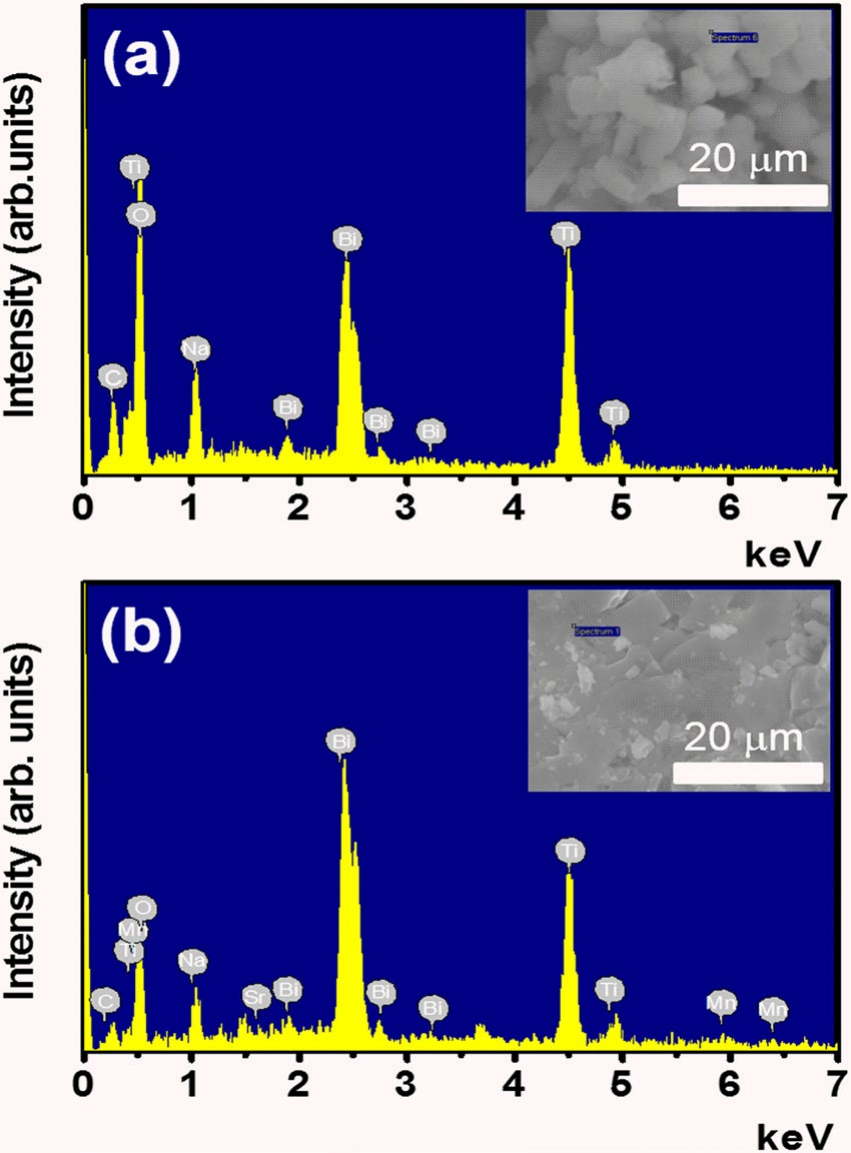
Energy dispersive X-ray spectra of (**a**) pure Bi_0.5_Na_0.5_TiO_3_ samples and (**b**) SrMnO_3-δ_-modified Bi_0.5_Na_0.5_TiO_3_ samples with 5 mol% SrMnO_3-δ_ as solid solution. Selection areas for composition element characterization are shown in the inset of each figure.

The chemical composition of pure Bi_0.5_Na_0.5_TiO_3_ and SrMnO_3-δ_-modified Bi_0.5_Na_0.5_TiO_3_ materials were confirmed, as shown in Fig. 1(a,b) for the energy dispersive X-ray spectra of pure and 5 mol% SrMnO_3−δ_-modified Bi_0.5_Na_0.5_TiO_3_ compounds, respectively, wherein which the selected area for the characterisation element was shown in the inset of each figure. The results showed that the all expectations of elements such as Bi, Na, Ti and O were obtained in pure Bi_0.5_Na_0.5_TiO_3_ as shown in Fig. 1(a). The EDX spectra of SrMnO_3−δ_-modified Bi_0.5_Na_0.5_TiO_3_ compounds showed the spectral addition elements such as Sr and Mn tailoring with elements Bi, Ti, Na and O host Bi_0.5_Na_0.5_TiO_3_ compounds.

### Surface morphologies

**Figure 2:**
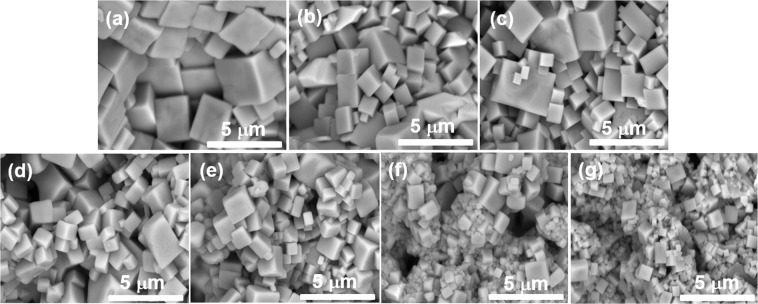
Surface morphology of (**a**) pure Bi_0.5_Na_0.5_TiO_3_, and (**b**–**f)** SrMnO_3−δ_-modified Bi_0.5_Na_0.5_TiO_3_ with 0.5, 1, 3, 5, 7, and 9 mol.%, respectively.

The solid solution of SrMnO_3−δ_ into host Bi_0.5_Na_0.5_TiO_3_ materials resulted in modification of the surface morphologies of samples where the surface morphologies were exhibited inhomogeneous as increasing the SrMnO_3−δ_ amounts solution. Figure 2(a–g) show the surface morphology of pure Bi_0.5_Na_0.5_TiO_3_ samples and SrMnO_3−δ_-modified Bi_0.5_Na_0.5_TiO_3_ samples with 0.5, 1, 3, 5, 7, and 9 mol%, respectively. The surface morphology of pure Bi_0.5_Na_0.5_TiO_3_ materials exhibited cubic-like shape with grain size of approximately 3–4 μm, as shown in Fig. 2(a). The minimal addition of 0.5 and 1 mol% SrMnO_3−δ_ into host Bi_0.5_Na_0.5_TiO_3_ materials resulted in inhomogeneous structure with grain size ranging from 1 μm to 4 μm, as shown in Fig. 2(b,c), respectively. Further addition of SrMnO_3−δ_ into host Bi_0.5_Na_0.5_TiO_3_ materials with SrMnO_3-δ_ up to 9 mol% as solid solution reduced the grain size and inhomogeneous distribution of the grain in a wide range from several hundred nanometers to few micrometers, as shown in Fig. 2(d–g). Normally, the presentation of impurities near the grain boundaries resulted in decreasing their mobility substantially as densification occurs. Therefore, small grain size is formed because of the reduction in the mobility of the grain boundary weakens the mass transport, resulting in obviously inhibited grain growth^32,33^. However, the grain size possibly increased because the presentation of oxygen vacancies is beneficial to mass transport during sintering^32,33^. Therefore, we suggested that the combination of both impurities and oxygen vacancy parameter affected the grain growth of SrMnO_3−δ_-modified Bi_0.5_Na_0.5_TiO_3_ samples, wherein Sr and Mn impurities cations showed inhibited grain growth, whereas oxygen vacancies promoted grain growth.

### Room temperature structure

**Figure 3:**
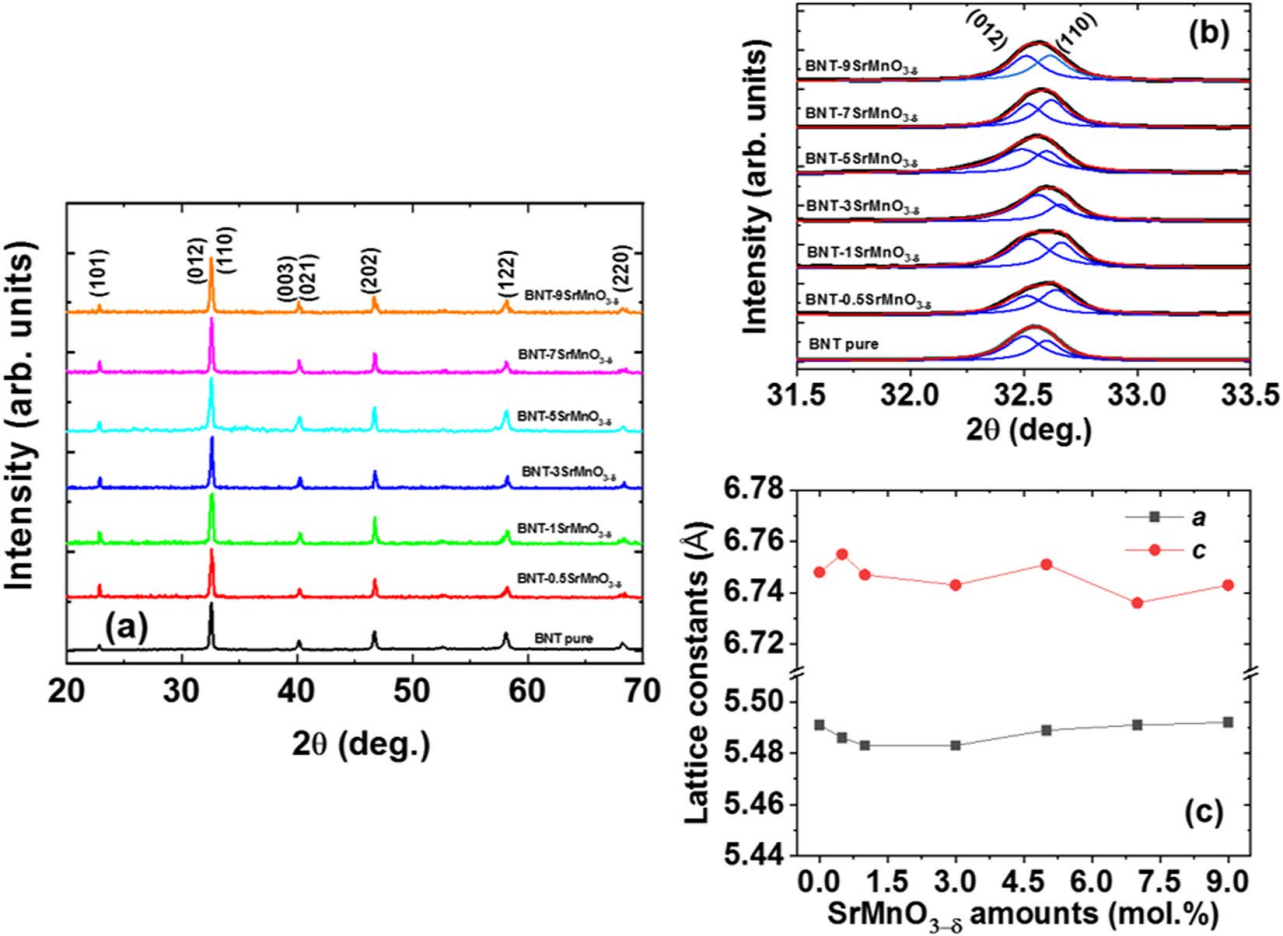
(**a**) X-ray diffraction spectra of pure Bi_0.5_Na_0.5_TiO_3_ and SrMnO_3-*δ*_-modified Bi_0.5_Na_0.5_TiO_3_ samples with various concentrations of SrMnO_3-*δ*_, (**b**) magnification and deconvolution of X-ray diffraction spectra of pure Bi_0.5_Na_0.5_TiO_3_ and SrMnO_3-*δ*_*-*modified Bi_0.5_Na_0.5_TiO_3_ samples in the 2*θ* range of 31°–35° with various concentrations, and (**c**) the dependent of lattice parameter of pure Bi_0.5_Na_0.5_TiO_3_ and SrMnO_3-*δ*_*-*modified Bi_0.5_Na_0.5_TiO_3_ samples as function of SrMnO_3-δ_ as solid solution.

The structural studied in SrMnO_3−δ_-modified Bi_0.5_Na_0.5_TiO_3_ compound with SrMnO_3−δ_ concentration up to 9 mol.% were provided that the SrMnO_3−δ_ were well solute into host Bi_0.5_Na_0.5_TiO_3_ crystal. Figure 3(a) shows the XRD of pure Bi_0.5_Na_0.5_TiO_3_ samples and SrMnO_3-δ_-modified Bi_0.5_Na_0.5_TiO_3_ samples with various SrMnO_3−δ_ concentrations. The peak position and relative intensity of peaks of pure Bi_0.5_Na_0.5_TiO_3_ samples were indexed as perovskite structure with rhombohedral symmetry. No diffraction peaks of SrMnO_3−δ_ materials were observed, as shown in the XRD spectra. Besides, the impurity phase was not obtained in the XRD spectra under the resolution of XRD method. The results indicated that the SrMnO_3−δ_ phase was a good solid solution for host Bi_0.5_Na_0.5_TiO_3_ materials. The influence of solid solution SrMnO_3−δ_ on the lattice of Bi_0.5_Na_0.5_TiO_3_ materials is shown in Fig. 3(b), wherein the XRD spectra were enlarged in 2θ range from 30°–35° for (012)/(110) peaks. The peak position was overlapped together. Therefore, the peak positions were distinguished using the Lorentzian fitting with r-square of over 0.99. The peak position clearly shifted to a high angle for 3 mol% SrMnO_3−δ_-modified Bi_0.5_Na_0.5_TiO_3_ materials. Furthermore, addition of SrMnO_3−δ_ into Bi_0.5_Na_0.5_TiO_3_ materials as solid solution which SrMnO_3-δ_ over 3 mol.% were resulted in the shrinkage of the lattice parameter as evidence by the shifting of the peak position to a low diffraction angle. The distortion of the lattice parameter was strong evidence for the random substitution of Sr and Mn cations into the host lattice of Bi_0.5_Na_0.5_TiO_3_ materials. The lattice constant of pure Bi_0.5_Na_0.5_TiO_3_ materials and SrMnO_3–δ_-modified Bi_0.5_Na_0.5_TiO_3_ materials as a function of SrMnO_3–δ_ concentration is calculated and presented in Fig. 3(c). The results further indicated that the lattice parameter of Bi_0.5_Na_0.5_TiO_3_ compounds was complex in distortion via addition of the different SrMnO_3−δ_ concentrations. The distorted lattice parameters were possibly understood when the radius of cations in host lattice was identified and compared with substitution impurities. The radius of Sr^2+^ (1.44 Å) cations is larger than that of average *A*-site (Bi^3+^/Na^+^) of 1.28 Å (Bi^3+^ (1.17 Å)/Na^+^ (1.39 Å))^34^. However, the complex substitution of Sr^2+^ cations for Bi^3+^ or Na^+^ cations in host lattice resulted in different behaviour, wherein Sr^2+^ cations substituted for Bi^3+^ cations generated the oxygen vacancies, whereas Sr^2+^ cations replacing for Na^+^ cations created the Na-vacancies^35^. Therefore, the Sr substitution in *A*-site was complex in distorted the lattice parameter. In addition, the Mn cations have multivalence states, e.g. as Mn^2+^, Mn^3+^ and Mn^4+^. Moreover, the spin state of each valence state influences the radius radii of cation Mn. Mn^2+^ cations have radii of 0.67 Å and 0.830 Å for low-spin and high-spin states, respectively^34^. The Mn^3+^ cations at low-spin and high-spin states have radii of 0.58 Å and 0.645 Å, respectively, whereas the Mn^4+^ cations with only high spin state have radii of 0.530 Å^34^. The Ti^4+^ cations in coordination number of VI have radii of 0.605 Å^34^. Therefore, if Mn cations exist at Mn^3+^ with low-spin state and Mn^4+^, then substitution for Ti-octahedral structure results in compressor lattice parameter and otherwise expands the lattice parameter of host Bi_0.5_Na_0.5_TiO_3_ materials. Thus, the distortion lattice parameter has a major influence on valence state and spin-state of Mn cations. However, the valence and spin state of Mn cations are very complex and strongly dependent on the chemical environment around the impurities of host materials and on the fabrication condition. Erdem *et al*. reported that Mn^2+^ and Mn^3+^ state existed in Bi_0.5_Na_0.5_TiO_3_-BaTiO_3_ materials^36^. Meanwhile, Li *et al*. reported that the mix Mn^2+^/Mn^4+^ valence states are obtained in Mn-doped Bi_0.5_Na_0.5_TiO_3_-BaTiO_3_-based material^37^. Hejazi *et al*. obtained the multivalence states of Mn such as Mn^2+^, Mn^3+^ and Mn^4+^ in Mn-doped Bi_0.5_Na_0.5_TiO_3_-based thin films^38^. Anthoniappen *et al*. reported that the Mn^3+^ possibly substituted at Ti-site, whereas Mn^2+^ was locally at the grain boundary^39^. Aksel *et al*. reported that the valence state of Mn changed from Mn^3+^ to Mn^2+^ while increasing the sintering temperature obtained in Mn-doped Bi_0.5_Na_0.5_TiO_3_ materials^40^. In addition, due to a lower valence state compared with Ti^4+^, the incorporation of Mn^2+^ and/or Mn^3+^ into the octahedral site of the structure produces excess negative charges, resulting thereby creating oxygen vacancies maintaining compensate for the maintenance of the overall electrical neutrality. Notably, the radius of oxygen vacancies (1.31 Å), which shrunk the lattice parameter, was smaller than that of oxygen anion (1.4 Å)^41^. The result indicated the XRD peak of host Bi_0.5_Na_0.5_TiO_3_ materials shifted after carrier SrMnO_3−δ_ was used as the solid solution, thereby providing evidence for the incorporation of Sr and Mn into the host lattice. In other words, the SrMnO_3−δ_ materials were good solid solutions in Bi_0.5_Na_0.5_TiO_3_ materials.

### The solute solution of SrMnO_3−δ_ into host Bi_0.5_Na_0.5_TiO_3_ materials was further confirmed by using Raman scattering studies

**Figure 4:**
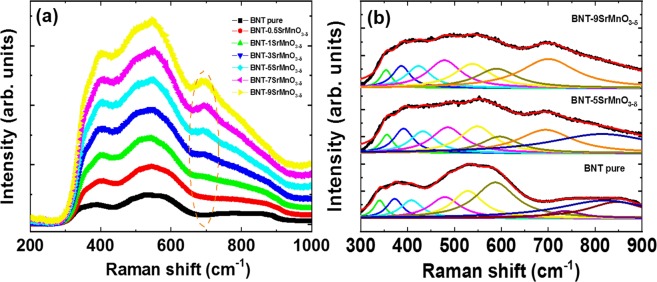
(**a**) Raman scattering spectra of pure Bi_0.5_Na_0.5_TiO_3_ and SrMnO_3-*δ*_-modified Bi_0.5_Na_0.5_TiO_3_ samples with various amounts of SrMnO_3-*δ*_, and (**b**) deconvolution peak position of pure Bi_0.5_Na_0.5_TiO_3_ and SrMnO_3-δ_-doped Bi_0.5_Na_0.5_TiO_3_ samples as solid solution with 5 and 9 mol% SrMnO_3-*δ*_^.^.

Figure 4(a) shows the Raman scattering spectra of pure Bi_0.5_Na_0.5_TiO_3_ and SrMnO_3−δ_-modified Bi_0.5_Na_0.5_TiO_3_ as solid solution at various SrMnO_3−δ_ concentrations. The pure Bi_0.5_Na_0.5_TiO_3_ sample exhibited broad band Raman scattering, which resulted from the random distribution of Bi and Na cations at *A*-site in perovskite structure^42^. However, the Raman scattering spectra of Bi_0.5_Na_0.5_TiO_3_ samples can be devised into three main regions in the wavenumber range of 300–1000 cm^−1^. The addition of SrMnO_3−δ_addition into Bi_0.5_Na_0.5_TiO_3_ materials caused to the occurrence of new vibration modes. We used the Lorentz fitting to estimate each vibration peak for pure Bi_0.5_Na_0.5_TiO_3_ and SrMnO_3−δ_-modified Bi_0.5_Na_0.5_TiO_3_ samples. The results of distinguished vibration modes of pure Bi_0.5_Na_0.5_TiO_3_ and SrMnO_3−δ_-modified Bi_0.5_Na_0.5_TiO_3_ samples with selected SrMnO_3−δ_ concentration are shown in Fig. 4(b). The nice vibration modes were obtained for pure Bi_0.5_Na_0.5_TiO_3_ samples, and this finding was consistent with the theoretical prediction of Niranjan *et al*.^42^. The addition of the vibration modes at approximately 670 cm^−1^ was obtained for SrMnO_3−δ_-modified samples, and the intensity of peak increased with the increase of increasing SrMnO_3−δ_ concentration. The appearance of new vibration modes was suggested for Mn substitution of Ti site to induce the MnO_6_ cluster vibration^43,44^. The band observed at approximately 528 cm^−1^ was relative to the breathing modes of TiO_6_ octahedral structure, which shifted to a high wavenumber for 5 mol% SrMnO_3−δ_ dopants then shifted back to low wavenumber for 9 mol% SrMnO_3−δ_ dopants. This result was consistent with the distorted XRD study for structural distortion of Bi_0.5_Na_0.5_TiO_3_ solid solution with SrMnO_3−δ_. The Bi/Na–O vibration at low vibration modes was not recorded due to the limitations of our experimental measurement setup. The structural XRD and Raman scattering study of SrMnO_3−δ_-modified Bi_0.5_Na_0.5_TiO_3_ samples indicated that Mn possible substituted for the Ti site.

### Optical properties

**Figure 5:**
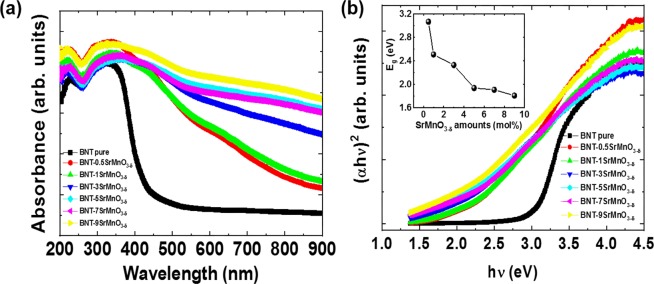
(**a**) Absorbance spectra of pure Bi_0.5_Na_0.5_TiO_3_ and SrMnO_3-*δ*_-modified Bi_0.5_Na_0.5_TiO_3_ samples with 0.5, 1, 3, 5, 7, and 9 mol.%, and (**b**) plot of (αhγ)^2^ values as a function of absorbance photon energy (hγ) for pure Bi_0.5_Na_0.5_TiO_3_ and SrMnO_3-*δ*_-modified Bi_0.5_Na_0.5_TiO_3_ samples with various SrMnO_3-*δ*_ amounts. Optical band gap energy as a function of SrMnO_3-*δ*_-modified Bi_0.5_Na_0.5_TiO_3_ samples as solid solution is shown the inset of (**b**).

The solute solution of SrMnO_3−δ_ into host Bi_0.5_Na_0.5_TiO_3_ materials results in reduction of optical band gap energy. Figure 5(a) shows the absorbance coefficient as function of absorption photon wavelength for pure Bi_0.5_Na_0.5_TiO_3_ and SrMnO_3−δ_-modified Bi_0.5_Na_0.5_TiO_3_ samples with various SrMnO_3−δ_ amounts. The single absorbance edge at approximately 400 nm with a small tail was obtained for pure Bi_0.5_Na_0.5_TiO_3_ samples. The small tail shown in the absorbance spectra of pure Bi_0.5_Na_0.5_TiO_3_ samples was related to self-defect and/or surface defect^45^. The addition of SrMnO_3−δ_-addition into host Bi_0.5_Na_0.5_TiO_3_ materials changed the optical properties of Bi_0.5_Na_0.5_TiO_3_ materials. The absorbance edge of SrMnO_3−δ_-modified Bi_0.5_Na_0.5_TiO_3_ samples tended to shift to high wavelength and was not clearly shown due to contribution of absorbance peaks of impurity cations. In addition, the various absorbance peaks were obtained in the absorbance spectra of SrMnO_3−δ_-modified Bi_0.5_Na_0.5_TiO_3_ samples, which were suggested for various transitions of the transition level energy of impurity cations. Bi_0.5_Na_0.5_TiO_3_ materials with direct transition, wherein in which the electronic band structures were constructed from Bi-6*s*, Ti-4*s* and O-2*p* for conduction band, and the bottom valence band mainly consisting of O-2*p*, Na-3*s* and Ti-3*d* orbitals were theoretically predicted^46,47^. Thus, we used Wood–Tauc method to estimate the value of optical band gap of pure Bi_0.5_Na_0.5_TiO_3_ and SrMnO_3−δ_-modified Bi_0.5_Na_0.5_TiO_3_ samples. The optical band gap *E*_*g*_ can be obtained from the intercept of (αhν)^1/n^ versus photon energy (hν), where α, ν and *h* are coefficient absorbance, wavelength and Plank constant, respectively. The band gap values were estimated with *n* = 1/2 for direct transition. The dependence of (αhν)^1/2^ to (hν) for pure Bi_0.5_Na_0.5_TiO_3_ and SrMnO_3−δ_-modified Bi_0.5_Na_0.5_TiO_3_ samples is shown in Fig. 5(b). *E*_g_ of pure Bi_0.5_Na_0.5_TiO_3_ was estimated at approximately 3.07 eV, which were consistent with the recently reported optical band gap of that materials in the range 3.00–3.14 eV^6,48^. SrMnO_3−δ_ addition into Bi_0.5_Na_0.5_TiO_3_ materials reduced the optical band gap to 1.81 eV for 9 mol% SrMnO_3−δ_. The details of dependent optical band gap values of Bi_0.5_Na_0.5_TiO_3_ as a function of SrMnO_3−δ_ amount solid solution into Bi_0.5_Na_0.5_TiO_3_ are shown in the inset of Fig. 5(b). The reduction of optical band gap of Bi_0.5_Na_0.5_TiO_3_ was consistent with the recently reported Mn- or Cr-doped Bi_0.5_Na_0.5_TiO_3_ materials, which resulted from the occurrence of local energy state of transition metal in the middle of the electronic band structure of Bi_0.5_Na_0.5_TiO_3_ materials^15,16^. In addition, the created oxygen vacancies caused by unbalance charges between Mn^2+/3+^ with Ti^4+^ and/or Sr^2+^ with Bi^3+^ were flexible enough to reduce the optical band gap, because the oxygen vacancy state was locally near the conduction band^49^. The substitution of Sr^2+^, possibly acting as a donor, for Na^+^ at *A*-site created the Na-site vacancies.

### The addition of SrMnO_3−δ_ into host Bi_0.5_Na_0.5_TiO_3_ materials as solid solution were suppressed the photoluminescence

**Figure 6:**
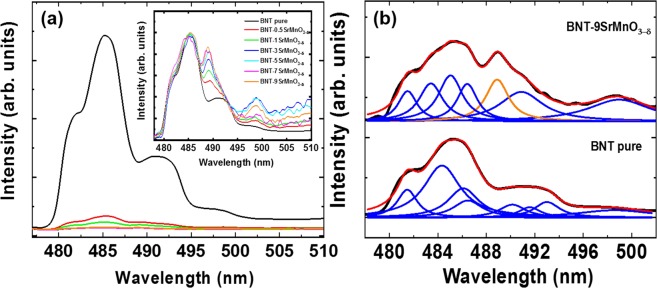
(**a**) Photoluminescence of pure Bi_0.5_Na_0.5_TiO_3_ and SrMnO_3-*δ*_-modified Bi_0.5_Na_0.5_TiO_3_ samples with various SrMnO_3-*δ*_ concentrations at room temperature, and (**b**) deconvolution photoluminescence peaks for pure Bi_0.5_Na_0.5_TiO_3_ and 9 mol% SrMnO_3-*δ*_-modified Bi_0.5_Na_0.5_TiO_3_ samples. Inset of (**a**) shows the comparison of the photoluminescence peak positions of pure Bi_0.5_Na_0.5_TiO_3_ and SrMnO_3-*δ*_-modified Bi_0.5_Na_0.5_TiO_3_ samples as solid solution with various concentrations of SrMnO_3-*δ*_ after substrate photoluminescence intensity to the unit.

Figure 6(a) shows the photoluminescence (PL) emission spectra of pure Bi_0.5_Na_0.5_TiO_3_ and SrMnO_3−δ_-modified Bi_0.5_Na_0.5_TiO_3_ samples at room temperature. The spectra of pure Bi_0.5_Na_0.5_TiO_3_ samples clearly showed a broad blue emission band within 476–510 nm relative with various transitions. The addition of SrMnO_3−δ_-addition into Bi_0.5_Na_0.5_TiO_3_ materials as solid solution decreased the intensity of PL emission. In addition, new emission peaks appearing at approximately 489 nm, and the intensity were increased with the increase of increasing SrMnO_3−δ_ amounts, as shown in the inset of Fig. 6(a) after the standard unit. We tried to distinguish each contribution PL peak via the Lorentz fitting. The results were shown in the examples for undoped Bi_0.5_Na_0.5_TiO_3_ and 9 mol% SrMnO_3−δ_-modified Bi_0.5_Na_0.5_TiO_3_ samples, as exhibited in Fig. 6(b) for down and up separation figures, respectively. The contribution of various PL peaks is unclear and requires further theoretical investigation. Normally, the PL of ferroelectric materials was not simply from band-to-band transition. The combination of photo-electron–hole pairs was difficult to combine due to the separation of the nature polarisation of electrical domain in materials. However, the surface states are often regarded as the dominant cause of luminescence in perovskites. A large number of unsaturated atoms exist on the surface of the perovskites, thereby forming localised levels within the forbidden gaps of the materials. Recently, Bac et al. suggested that the observation of PL of Bi_0.5_K_0.5_TiO_3_ materials was related to the disorder coupled with the tilt of TiO_6_-TiO_6_ adjacent octahedral structure, thereby resulting in structural distortion and generation of localised electronic levels above the valence band^45^. The re-appearance of new photoluminescent peaks was suggested reliving with the new vibration of MnO_6_-TiO_6_ or MnO_6_-MnO_6_ adjacent octahedral structure, as exhibited in the Raman scattering results. The replacement of Mn^2+/3+^ cations for Ti^4+^ cations at *B*-site and Sr^2+^ cation for Bi^3+^ cations at *A*-site created oxygen vacancies, which trapped the electron generated from absorbance photon energy and prevented the recombination of the electron–holes to generate photon, suppressing the PL intensity of SrMnO_3−δ_-modified Bi_0.5_Na_0.5_TiO_3_ samples. In addition, the substitution of Sr^2+^ cations for the resulting Na^+^ cations created the Na vacancies, which also acted as chapping for electrons, thereby reducing the combination of the photon-electron–hole pair.

### Magnetic properties

**Figure 7:**
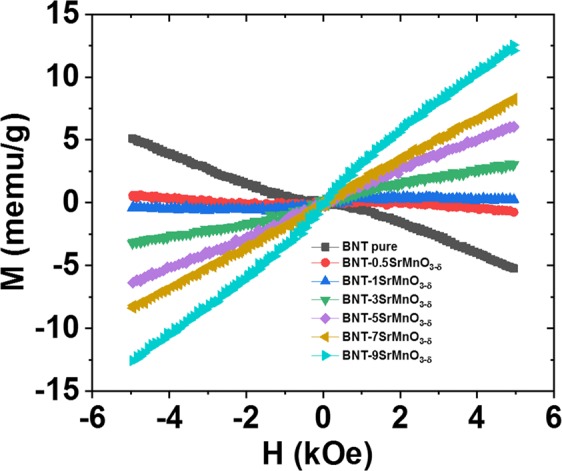
Magnetization as a function of applied magnetic field of pure Bi_0.5_Na_0.5_TiO_3_ samples SrMnO_3-*δ*_-modified Bi_0.5_Na_0.5_TiO_3_ samples at room temperature with various SrMnO_3-*δ*_ concentrations as solid solution.

The complex magnetic properties at room temperature of Bi_0.5_Na_0.5_TiO_3_ materials were obtained as function of SrMnO_3−δ_ solute solution. Figure 7 show the magnetization as function of applied magnetic field (*M–H*) for pure Bi_0.5_Na_0.5_TiO_3_ samples and SrMnO_3−δ_-modified Bi_0.5_Na_0.5_TiO_3_ sample with various SrMnO_3−δ_ amounts from 0.5 to 9 mol.%. The pure Bi_0.5_Na_0.5_TiO_3_ materials exhibited the anti-S-shape in *M–H* curves, which resulted from the combination of diamagnetic and weak ferromagnetic properties. The diamagnetic property of Bi_0.5_Na_0.5_TiO_3_ materials behaviour originated from the empty state of Ti^4+^ cations with 3*d*° states^15,16^. The weak-ferromagnetism observation in pure Bi_0.5_Na_0.5_TiO_3_ materials was possibly related with self-defect and/or surface effects, which were well explained by the first-principle theoretical prediction and experimental achievement^15–17,50^. The anti-*S*-shape in *M–H* curve was obtained for 0.5 mol% SrMnO_3−δ_ solid solution into Bi_0.5_Na_0.5_TiO_3_ sample. The saturation trend in magnetisation for SrMnO_3−δ_-doped Bi_0.5_Na_0.5_TiO_3_ with 1 mol% SrMnO_3−δ_. Further addition of the SrMnO_3−δ_ concentration into Bi_0.5_Na_0.5_TiO_3_ materials resulted in unsaturation of magnetisation with low applied magnetic field. The slight addition of SrMnO_3−δ_ into Bi_0.5_Na_0.5_TiO_3_ induced the ferromagnetism due to the interaction of Mn^2+/3+^ through oxygen vacancies (□)^49^. Furthermore, the solid solution of SrMnO_3−δ_ into Bi_0.5_Na_0.5_TiO_3_ samples enhanced the ferromagnetic ordering due to several favourable Mn^2+/3+^-□-Mn^2+/3+^. In addition, the vacancies of such Na-vacancies, which causes the substitution of Sr^2+^ for Na^+^ at *A*-site in perovskite structure, influenced the ferromagnetism in samples^14,17^. Moreover, if Sr^2+^ cations substituted for Bi^3+^ cations were possible resulted in changing the valence state of Ti^4+^ to Ti^3+^ state cause of enhancement the number of oxygen vacancies^35^. Our recently predicted that the Ti^3+^-defects state in Bi_0.5_K_0.5_TiO_3_ materials was strong induced the ferromagnetism^51^. However, the unsaturation in magnetisation as a function of low applied magnetic field via further addition of SrMnO_3−δ_ (over 3 mol%) into Bi_0.5_Na_0.5_TiO_3_ materials was possibly related to the isolation of Mn cations, which favoured of paramagnetic property or/and interaction of polaron (Mn^2+/3+^-□-Mn^2+/3+^) vs. (Mn^2+/3+^-□-Mn^2+/3+^), which favoured the antiferromagnetic-like materials^52^. The magnetisation of 9 mol%-doped Bi_0.5_Na_0.5_TiO_3_ samples was achieved at approximately 12.5 memu/g at room temperature, which was greatly enhanced compared with pure Bi_0.5_Na_0.5_TiO_3_ materials^15,16^. The values were also larger than that of Mn-, Fe-, Cr- and Co-doped Bi_0.5_Na_0.5_TiO_3_ materials^15,16,18,19^. Thus, we suggested that the co-modification at *A*-site and *B*-site via alkalize and transition metals, respectively, displayed higher performance magnetic properties than single transition metal-doped Bi_0.5_Na_0.5_TiO_3_ materials. However, the role of *A*-site-modification on the magnetic properties of Bi_0.5_Na_0.5_TiO_3_-dope-material with transition metal needs further theoretical calculated investigation.

## Discussion

The new system Bi_0.5_Na_0.5_TiO_3_-SrMnO_3−δ_ solid solution materials were fabricated via sol–gel method. X-ray diffraction and Raman scattering were used to study the structure of pure Bi_0.5_Na_0.5_TiO_3_ and SrMnO_3−δ_-modified Bi_0.5_Na_0.5_TiO_3_ materials with various SrMnO_3-δ_ amount, providing that all samples followed the crystal structural symmetry of host rhombohedral structure of Bi_0.5_Na_0.5_TiO_3_ materials. This phenomenon indicated that the SrMnO_3−δ_ materials were good solid solution in host Bi_0.5_Na_0.5_TiO_3_ crystal structure. The Sr and Mn cations were diffused to random incorporation with host lattice of Bi_0.5_Na_0.5_TiO_3_ crystal to form as solid solution, resulting in complex-distorted structure. The random distribution of Sr^2+^ cations into *A*-site of Bi_0.5_Na_0.5_TiO_3_ materials was possibly different, indicating that Sr^2+^ cations substituted for Bi^3+^ cations generate the oxygen vacancies, whereas the Sr^2+^ cations replaced for Na^+^ create the Na vacancies. The presentation of complex defects in Bi_0.5_Na_0.5_TiO_3_ materials during solid solution of SrMnO_3−δ_ reduced the optical band gap values from approximately 3.07 eV to 1.18 eV for 9 mol% SrMnO_3−δ_ solid solution. The absorbance spectroscopy of SrMnO_3_-modified Bi_0.5_Na_0.5_TiO_3_ materials exhibited the multi-absorbance peaks in visible absorbance range, presenting the multivalence state of Mn cations, such as Mn^2+^, Mn^3+^, and Mn^4+^. Thus, the modified *B*-site by multivalence state of Mn cations also possibly exhibited the different interactions, wherein the Mn^2+/3+^ cation interaction through the oxygen vacancies (Mn^2+/3+^-□-Mn^2+/3+^) resulted in ferromagnetic ordering, whereas Mn^4+^ cation interaction through oxygen (Mn^4+^-O^2−^-Mn^4+^) was favourable to antiferromagnetic ordering. Mn cation isolates incorporated with host lattice displayed the paramagnetic behaviour. The possible antiferromagnetic-like structure started to occur when the Mn cations were rich enough to bind together the superinteraction of pair (Mn^2+/3+^-□-Mn^2+/3+^) vs. (Mn^2+/3+^-□-Mn^2+/3+^). Therefore, by controlling the SrMnO_3−δ_ concentration doping in host lattice Bi_0.5_Na_0.5_TiO_3_ materials, the magnetic properties of Bi_0.5_Na_0.5_TiO_3_ materials were tuned from compensationof diamagnetic and weak ferromagnetic property of pure materials to typical ferromagnetism behaviour and at the end of combination of paramagnetism/antiferromagnetism-like versus ferromagnetism with the increase of the SrMnO_3−δ_ amount solid solution into host Bi_0.5_Na_0.5_TiO_3_ materials. We expected that co-modification at the *A*-site and *B*-site in lead-free ferroelectric perovskite *AB*O_3_ materials via alkali earth and transition metals, respectively, resulted in great enhancement of the ferromagnetism than that of contribution of self-defect and/or surface effects, or by using single transition metal dopants. We also expected that our method opened the new way to develop injection ferromagnetism in lead-free ferroelectric materials, such as BaTiO_3_-based and (K,Na)NbO_3_-based family, by using solid solution method. The observation of tuneable magnetic and optical properties of lead-free ferroelectric material was promising for application to green electronic devices.

## Methods

### Sample preparation

The pure Bi_0.5_Na_0.5_TiO_3_ compounds were fabricated from material source of bismuth nitrate pentahydrate (Bi(NO_3_)_3·_5H_2_O), sodium nitrate (NaNO_3_) and tetraisopropoxytitanium (IV, C_12_H_28_O_4_Ti). The Bi(NO_3_)_3_·5H_2_O and NaNO_3_ were weighed and distinguished in acetic acid and de-ions water H_2_O (*V*_H2O_:*V*_CH3COOH_ = 5: 1). The acetylacetone was added dropwise before the addition of C_12_H_28_O_4_Ti. The solution was magnetically stirred at approximately 3 h and heated followed by at100 °C to prepare the gel. The (*x*)SrMnO_3−δ_ + (1−*x*)Bi_0.5_Na_0.5_TiO_3_ (*x* = 0.5, 1, 3, 5, 7, 9 mol%) compounds were fabricated by using a similar method of fabrication with pure Bi_0.5_Na_0.5_TiO_3_. However, the starting materials were Sr(NO_3_)_2_ and a solution of Mn(NO_3_)_3_ (60%). These materials were weighed, and the solution was added by estimating the dopant concentrations. The gels were ground and annealed for 5 h at 800 °C and then naturally cooled to room temperature. Extra sodium was added at approximately 50 mol% to prevent sodium loss during gelling and annealing processing^24,25^.

### Sample characterization

The surface morphology and presentation of the elements in samples was measured by Energy Dispersive X-ray analysis (EDX, S-4800 Hitachi). The chemical composition of pure Bi_0.5_Na_0.5_TiO_3_ samples and SrMnO_3−δ_-modified Bi_0.5_Na_0.5_TiO_3_ compounds was further confirmed by using an electron probe micro-analyzer (EPMA, Shimadzu EPMA 1600). The sample powder was ground for characterisation via X-ray diffraction (XRD, Brucker D8 Advance) and Raman scattering (with a 475 nm LASOS laser and a DU420A-Oe defector) to analyse the crystal structure and vibration mode of the atom, respectively. The absorbance spectroscopy and photoluminescent properties of pure and SrMnO_3−δ_-modified Bi_0.5_Na_0.5_TiO_3_ compounds were measured by using Ultraviolet–Visible spectroscopy (UV–Vis, Jasco V-670) and photoluminescence (exciter with 475 m LASOS laser and a DU420A-Oe defector), respectively. The magnetic properties were studied using a Vibrating Sample Magnetometry (VSM, Lakeshore 7404) at room temperature, respectively. Finally, we used X-ray photoelectron spectroscopy (XPS, Thermofisher, a twin anode X-ray source (Al Kα, hν =1686.6 eV) gun and monochromatic gun) to determine the valence state of the cations in the pure Bi_0.5_Na_0.5_TiO_3_ and 9 mol.% SrMnO_3__-δ_-modified Bi_0.5_Na_0.5_TiO_3_ samples, as shown in Figure S1 and Figure S2, respectively, in suplimental data. The X-ray diffraction peaks, Raman scattering peaks, XPS peaks and photoluminescence peaks were distinguished by using Lorentzian fitting with r-square over 0.99.

## Supplementary information

Structure, optical and magnetic properties of new Bi0.5Na0.5TiO3- SrMnO3−δ⎕ solid solution materials
