# Author Correction: Structure, optical and magnetic properties of new Bi_0.5_Na_0.5_TiO_3_- SrMnO_3−δ_ solid solution materials

**DOI:** 10.1038/s41598-020-58158-5

**Published:** 2020-01-22

**Authors:** Dang Duc Dung, Nguyen The Hung, Dorj Odkhuu

**Affiliations:** 1 Department of General Physics, School of Engineering Physics, Ha Noi University of Science and Technology, 1 Dai Co Viet road, Ha Noi, Viet Nam; 2 Department of Physics, Faculty of Basic and Fundamental Sciences, Viet Nam Maritime University, 484, Lach Tray street, Hai Phong city, Viet Nam; 3 Department of Physics, Incheon National University, Incheon, 22012 Republic of Korea

Correction to: *Scientific Reports* 10.1038/s41598-019-54172-4, published online 03 December 2019

The Acknowledgements section in this Article is incomplete.

“This work was financially supported by The Ministry of Science and Technology, Viet Nam, under project number ĐTĐLCN.29/18.”

should read:

“This work was supported by The Ministry of Science and Technology, Viet Nam, under project number ĐTĐLCN.29/18. This research was also supported by the Future Materials Discovery Program through the National Research Foundation of Korea (NRF) funded by the Ministry of Science and ICT (Grant No. 2016M3D1A1027831).”

