# Supplementary material for: Structure, optical and magnetic properties of new Bi0.5Na0.5TiO3- SrMnO3−δ solid solution materials
Source: Sci Rep. 2019 Dec 3;9:18186. doi: 10.1038/s41598-019-54172-4 (PMC6890638; doi:10.1038/s41598-019-54172-4)
Supplement: Supplementary file 1 — Structure, optical and magnetic properties of new Bi0.5Na0.5TiO3- SrMnO3−δ⎕ solid solution materials [file 41598_2019_54172_MOESM1_ESM.doc]

**Supporting information**

**Structure, optical and magnetic properties of new Bi_0.5_Na_0.5_TiO_3_- SrMnO_3−δ_ solid solution materials**

Dang Duc Dung^1,*^, Nguyen The Hung^1,2^, and Dorj Odkhuu^3,+^

*^1^Department of General Physics, School of Engineering Physics, Ha Noi University of Science and Technology, 1 Dai Co Viet road, Ha Noi, Viet Nam*

*^2^**Department of Physics, Faculty of Basic and Fundamental Sciences, Viet Nam Maritime University, 484 Lach Tray street, Hai Phong city, Viet Nam*

*^3^Department of Physics, Incheon National University, Incheon 22012, Republic of Korea*

Corresponding author:

Dang Duc Dung, e-mail: [dung.dangduc@hust.edu.vn](mailto:dung.dangduc@hust.edu.vn), and Dorj Odkhuu, e-mail: [odkhuu@inu.ac.kr](mailto:odkhuu@inu.ac.kr)

**Supplemental**.


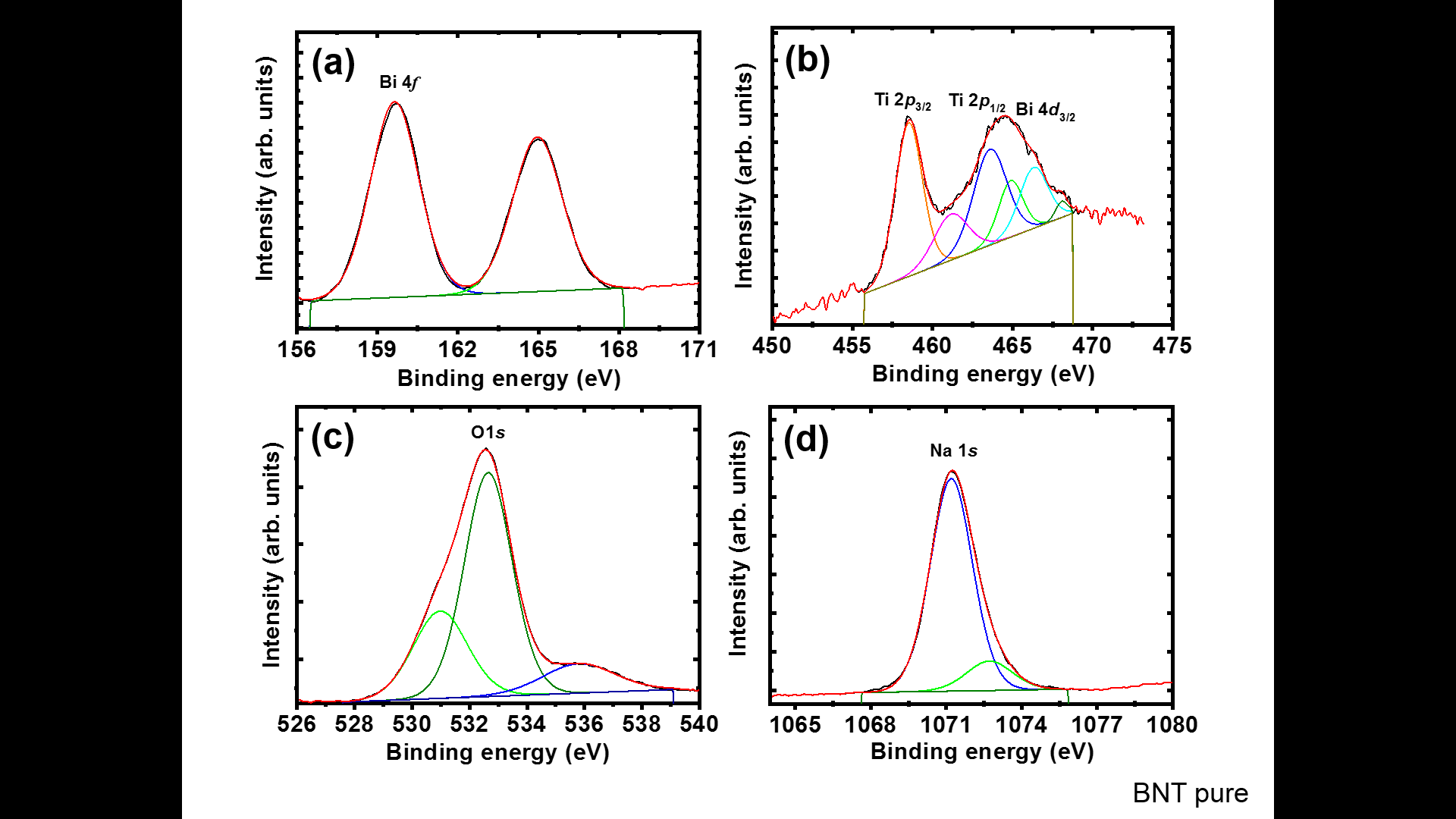


Figure S1. XPS spectra of pure Bi_0.5_Na_0.5_TiO_3_ samples: (a) Bi 2*p*; (b) Ti 2*p*; (c) O 1s; and (d) Na 1*s* elements.


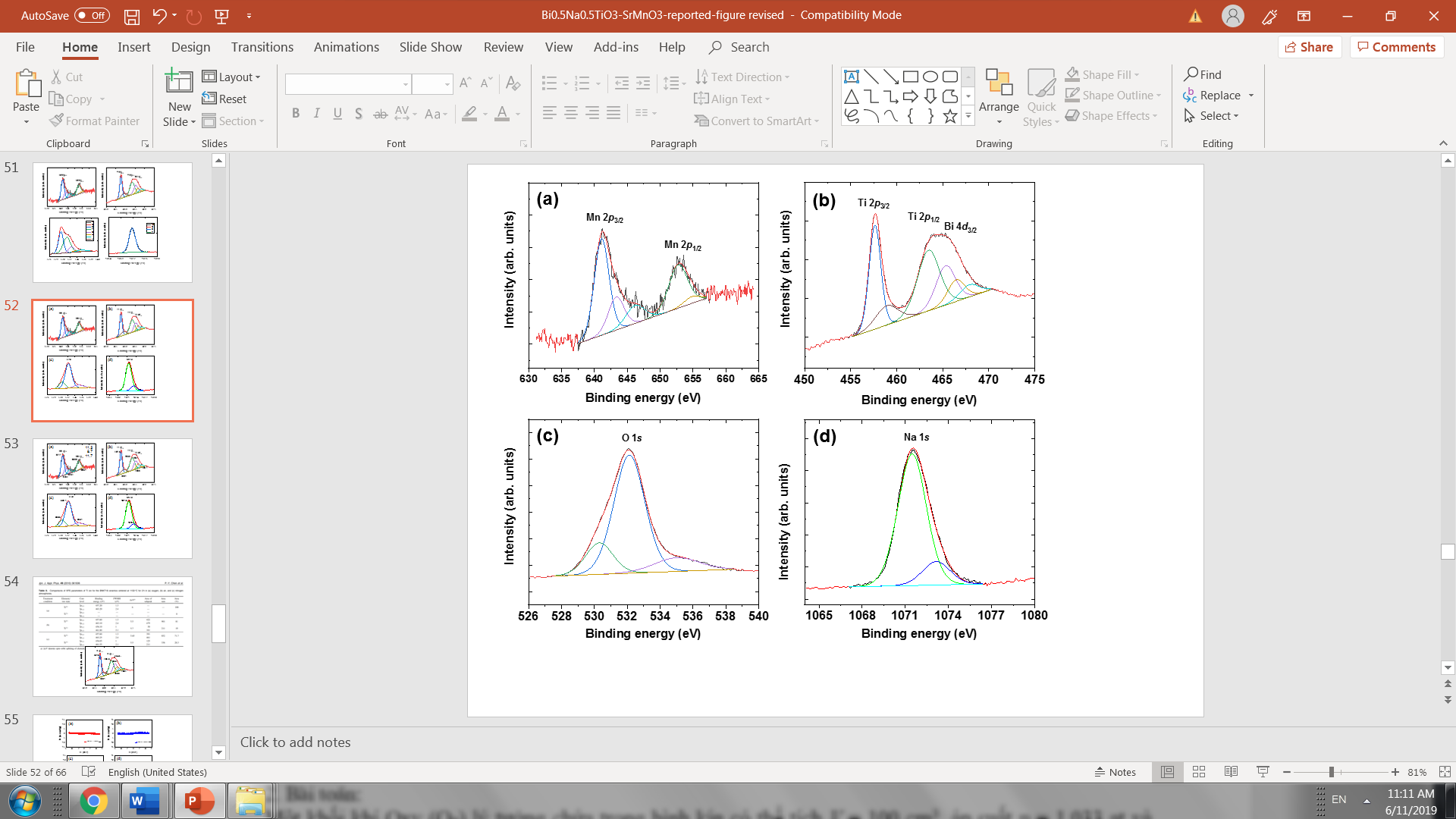


Figure S2. XPS spectra of SrMnO_3-δ_-doped Bi_0.5_Na_0.5_TiO_3_ samples with 9 mol% SrMnO_3-δ_ solid solution: (a) Mn 2*p*; (b) Ti 2*p*; (c) O 1s and (d) Na 1*s* elements.
